# Enteral Nutrition Is Associated with a Distinct Gut Microbiome Composition and Fermentation Capacity Profile After Acute Colonic Injury in Rats

**DOI:** 10.3390/ijms27177867

**Published:** 2026-09-02

**Authors:** Samat Kozhakhmetov, Elizaveta Vinogradova, Shynggys Sergazy, Laura Chulenbayeva, Alibek Kossumov, Artur Kovenskiy, Nurislam Mukhanbetzhanov, Gulnazym Ospankulova, Saule Saduakhasova, Dina Khamitova, Svetlana Kamanova, Dana Toimbayeva, Bakhyt Shaimenova, Begzhan Kalemshariv, Daulet Aitmukhanbetov, Almagul Kushugulova

**Affiliations:** 1 Laboratory of Microbiome, Center for Life Sciences, National Laboratory Astana, Nazarbayev University, 53 Kabanbay Batyr Ave., Block S1, Astana Z05H0P9, Kazakhstanakushugulova@nu.edu.kz (A.K.); 2 Kazakhstan Society of Human Microbiome Researchers, Astana Z05H0P9, Kazakhstan; 3 Innovative Center ArtScience, Astana Z11F5A9, Kazakhstan; 4 Interdisciplinary Sports Research, Center for Genetics and Life Sciences, Sirius University of Science and Technology, 1 Olympic Ave., Sirius Federal Territory 354340, Russia; 5 Victus Pharm, Astana Z19Y9B3, Kazakhstan; 6 Department of Food Technology and Processing Products, Technical Faculty, Saken Seifullin Kazakh Agrotechnical University, Zhenis Avenue, 62, Astana Z11F9K5, Kazakhstan; saulesaduahasova5@gmail.com (S.S.); kamanovasveta@mail.ru (S.K.); bio.dana@mail.ru (D.T.); bshaymenova@mail.ru (B.S.); begjan.ae@gmail.com (B.K.); 7 Regional Technological Park QazAgroOrganic LLP, Saken Seifullin Kazakh Agrotechnical University, Zhenis 62b, Astana Z11F9K5, Kazakhstan; 8 Department of Biology, School of Sciences and Humanities, Nazarbayev University, Kabanbay batyr 53, Astana Z05H0P9, Kazakhstan

**Keywords:** enteral nutrition, gut microbiome, dextran sulfate sodium (DSS), acute colonic injury, metagenomic next-generation sequencing (mNGS), *Lactobacillales*, lactate, microbiome functional profiling, rats

## Abstract

Enteral nutrition (EN) is known to promote mucosal healing in inflammatory bowel disease, and multi-omics data suggest that the gut microbiome mediates its therapeutic effects. However, the impact of EN and its components on the gut community during recovery from acute epithelial injury remains incompletely understood. We used whole-genome metagenomic sequencing to investigate the effect of an EN formula based on extruded amaranth flour and pea protein on the gut microbiome in a dextran sulfate sodium (DSS) rat model of acute colonic injury. Three groups were compared, as follows: an unchallenged control (*n* = 9) with standard chow, a colonic injury (5% DSS; *n* = 9) group with standard chow, and a colonic injury (5% DSS; *n* = 9) group with EN. Injury was confirmed histologically (median MCHI score was 2, indicating epithelial damage without inflammation). DSS caused significant weight loss. Animals receiving EN regained baseline weight faster, by day 14, whereas animals on standard chow achieved recovery only by day 21. Differences in energy intake should be further investigated to validate the effect of EN on body weight recovery. At day 21, both injury groups demonstrated higher relative abundances of *Bacteroidaceae* and *Erysipelotrichaceae*, including the mucin-degrader *Allobaculum mucilyticum*, compared with the control group. Conversely, *Lactobacillus* abundance, notably *Lactobacillus acidophilus*, was higher in the EN group than in both other groups, as was the inferred capacity for lactate-producing fermentation. These findings suggest that EN is associated with a distinct microbial composition and inferred metabolic profile during the post-injury period, with lactobacilli as one of the potential mediators of its effects.

## 1. Introduction

Enteral nutrition (EN), particularly exclusive enteral nutrition (EEN), has demonstrated efficacy in achieving clinical remission and mucosal healing in inflammatory bowel disease (IBD); however, its mechanisms of action remain incompletely understood [1]. Historically, the therapeutic effects of EN were ascribed to direct cellular nutrient utilization. More recently, accumulating multi-omics evidence has suggested that the gut microbiome plays a substantial role in mediating the therapeutic effects of EN.

Support for this hypothesis has emerged from several dietary intervention studies. In particular, prospective dietary intervention cohort data have indicated that EN actively shifts the microbiome functional profile of patients towards a healthier metabotype characterized by low *Pseudomonadota* (formerly *Proteobacteria*) abundance and high potential for short-chain fatty acids (SCFAs) synthesis pathways, suggesting correction of functional dysbiosis [2]. Another study further demonstrated that while EN is highly effective at inducing remission, the resulting taxonomic shifts are highly personalized and volatile, suggesting that it is changes in the microbiome metabolite profile that contribute to EN therapeutic efficacy [3]. Further research demonstrated that pre-treatment gut microbial metabolite signatures can predict response to EN, highlighting patient-specific responses to dietary changes mediated by gut microbiome [4]. Moreover, metabolomic profiling of fecal samples from IBD patients before and after EN treatment highlighted induction of significant metabolic changes, with EN appearing to modulate microbial metabolism, reducing gut tissue injury [5]. Together, these data suggest that EN efficacy is mediated by the gut microbiome, and that microbial metabolic capacity may serve as a sensitive indicator of recovery.

Despite these insights, EN protocols, remission rates, and formulae vary widely across the literature, and evidence regarding responses to specific EN components remains limited [1]. Consequently, our understanding of how EN and its constituents affect the gut community during recovery from acute epithelial injury remains incomplete, underscoring the need for further research. In addition, most experimental studies evaluating EN effects on the microbiome in intestinal injury have relied on 16S amplicon sequencing [3,4,5], which captures taxonomic structure but not genomic content, thereby limiting the assessment of functional microbiome capacity and representing a notable methodological gap. These limitations, together with an incomplete mechanistic understanding and limited data on responses to specific EN components, hinder both the broader clinical adoption of EN and the development of personalized nutritional approaches.

To further broaden our understanding of the gut microbiome’s role in EN-mediated recovery, in the present study we employed whole-genome metagenomic sequencing to investigate the effects of an EN formula administration based on extruded amaranth flour and pea protein on the taxonomic structure of the gut microbiome in a rat model of acute colonic injury, with the aim of identifying microbial and functional signatures associated with treatment response. The study included three groups, as follows: an unchallenged control group, a colonic injury group receiving standard chow, and a colonic injury group receiving EN.

In particular, in this study, to investigate the effects of EN on gut microbiome composition, a short-term dextran sulfate sodium (DSS)-induced epithelial damage [6] rat model with a spontaneous recovery phase was used. DSS is highly appropriate for investigating EN effects in relation to microbiome composition. In contrast to multi-week chronic colitis models, this design isolates the nutritional intervention specifically during active tissue repair, after the acute inflammatory burst has subsided but before the mucosal niche has fully stabilized. Preclinical studies show that inflammation-associated microbiota remain detectable even after a brief recovery period following DSS withdrawal, making this post-injury window a highly sensitive setting for testing how diet drives microbial reconstruction during healing [7], when the substrate composition of the diet acts as a powerful selective force on the surviving, destabilized microbial populations.

A polymeric EN formula composed of extruded amaranth flour and pea protein isolate, designed to exert a distinct ecological pressure on the recovering gut microbiota, was employed in this study. Plant-derived substrates are of particular relevance in this context, as follows: pea proteins have been demonstrated to modulate gut microbial community structure, and amaranth-based dietary interventions have been associated with favorable shifts in health-related taxa [8,9].

The aim of this study was to characterize the taxonomic composition and inferred functional capacity of the rat gut microbiome at the end of a 21-day recovery period following acute DSS-induced epithelial injury in animals receiving either standard chow or an EEN formula based on extruded amaranth flour and pea protein isolate.

## 2. Results

### 2.1. Experimental Design and General Group Characteristics

**Figure 1:**
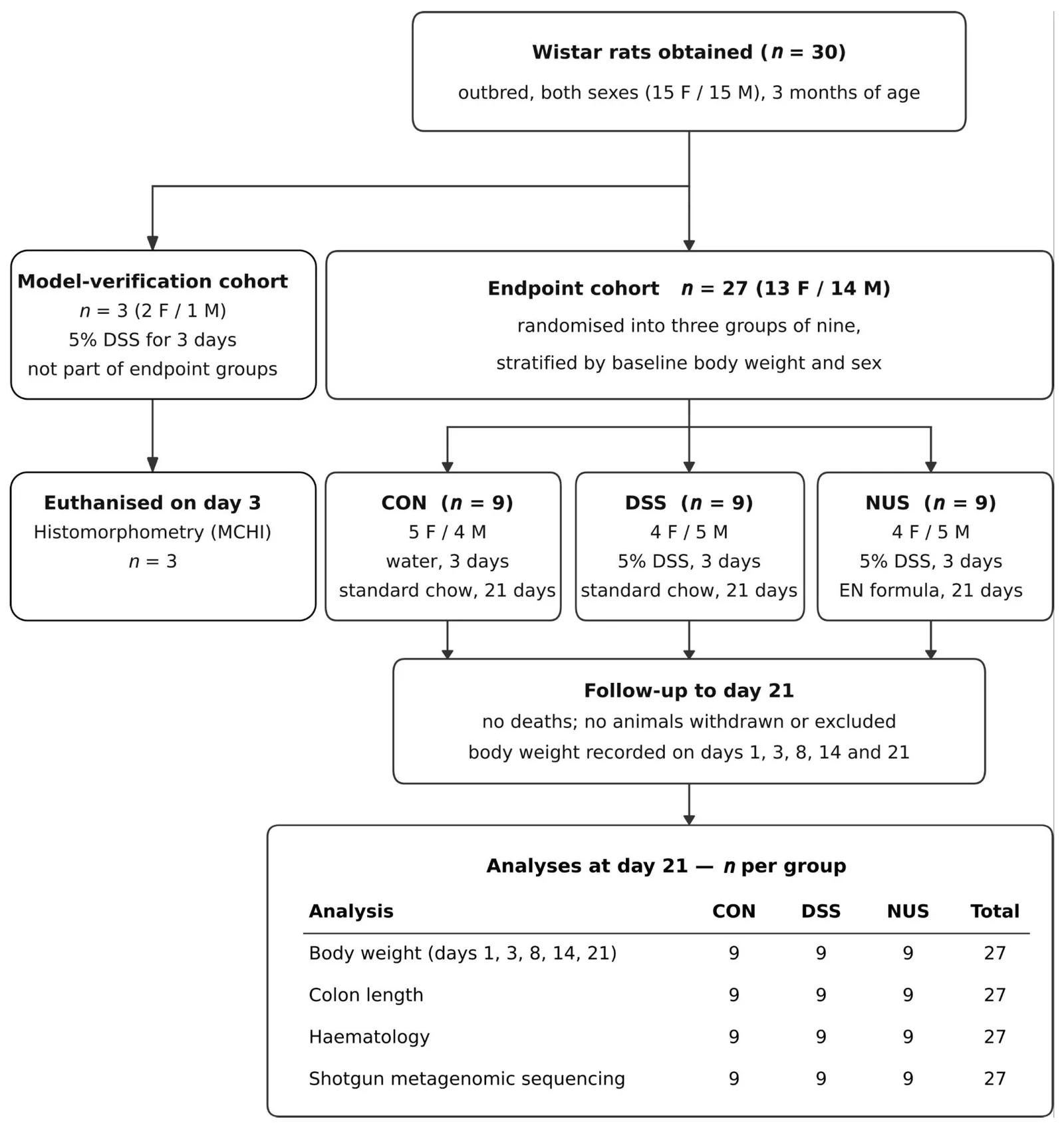
Animal flow diagram.

The effect of an exclusive enteral nutrition (EEN) formula based on extruded amaranth flour and pea protein isolate on the gut microbial community during the recovery phase following acute colonic injury was assessed in 27 animals, which were allocated into three groups, as follows: an unchallenged control (CON, *n* = 9), a colonic injury followed by standard chow (DSS, *n* = 9), and a colonic injury followed by enteral nutrition (EN) (NUS, *n* = 9). Acute injury was induced by administering a 5% dextran sulfate sodium (DSS) solution for 3 days. Subsequently, animals in the NUS group received the EEN formula as the sole caloric source for 21 days, whereas animals in the DSS group were fed standard chow. The unchallenged control group was not subjected to injury and served as the reference for normal taxonomic composition. Three additional animals were challenged with 5% DSS in parallel and euthanized on day 3 for histological verification of the injury model (Section 2.2); these animals were not part of the three endpoint groups and were excluded from all endpoint analyses. The animal flow diagram is shown in Figure 1.

### 2.2. Histomorphometric Assessment of the Colon on Day 3 Following DSS Administration

To confirm the occurrence of injury, three additional DSS-challenged animals (*n* = 3), which were not part of the CON, DSS, or NUS endpoint groups, underwent histomorphometric assessment of the colon 3 days after DSS administration using the Mouse Colitis Histology Index (MCHI) [10]. The samples exhibited focal and diffuse desquamation of the superficial mucosal epithelium, with areas of exposed lamina propria, denudation, and moderate flattening of crypts, and a reduced number of goblet cells due to acute cytolysis; mucosal architecture was partially disrupted. The cellular composition of the lamina propria, submucosa, and muscular layers showed no pathological changes, as follows: no signs of leukocytic, neutrophilic, or lymphocytic infiltration, crypt abscesses, lymphoid follicle hyperplasia, or compensatory epithelial proliferation were observed. The changes predominantly affected the epithelial–cryptal component (reduced crypt density, occasional ulcerations) in the absence of inflammatory infiltration across all three samples; the median total MCHI score was 2, corresponding to minimal tissue damage without an inflammatory component. The absence of infiltration and compensatory epithelial proliferation confirmed that the model reproduced acute toxic epithelial injury of the colon rather than chronic colitis.

### 2.3. Body Weight Dynamics Following DSS Administration

DSS administration induced a significant decrease in body weight in both the DSS and NUS groups (Table 1).

The maximum reduction was achieved on day 8, reaching −5.71% (interquartile range (IQR): −6.59; −4.87, *q* < 0.01) from baseline in the DSS group and −6.81% (IQR: −7.20; −6.39, *q* < 0.01) from baseline in the NUS group. Recovery to baseline was observed at day 21 in the DSS group (+0.51% (IQR: −2.9; 2.53), compared to baseline, *q* = 0.91), whereas the NUS group recovered earlier, by day 14 (+1.78% (IQR: 1.58; 1.93), compared to baseline, *q* < 0.01). Body weight dynamics differed significantly between groups across the measured time points (*q* < 0.001). However, daily caloric intake was not controlled, and thus differences in energy intake could not be ruled out as an explanation for the observed weight recovery; further investigation is therefore needed to validate the effect of enteral nutrition on body weight recovery.

### 2.4. Colon Length at the End of the Observation Period (Day 21)

Colon length on day 21 was as follows: CON 16.2 cm (IQR: 14.5; 17.3), DSS 15.3 cm (IQR: 14.5; 16.1), and NUS 14.4 cm (IQR: 14.2; 15.0). No significant differences were observed (Kruskal–Wallis test, *p* = 0.41). The absence of shortening is consistent with the acute injury model rather than with established colitis, although colon length is an indirect measure and does not itself demonstrate mucosal repair.

### 2.5. Hematological Parameters at the End of the Observation Period (Day 21)

Hematological profiles at the end of the observation period (day 21) did not differ substantially across groups (Table 2).

Lymphocyte counts were lower in both DSS-exposed groups compared with the control (*q* < 0.05), whereas granulocyte proportions were slightly elevated (*q* < 0.05). Erythrocyte parameters showed no differences between groups (*q* > 0.05). Lastly, the percentage of large platelets was higher in the DSS group on standard chow relative to all other groups (*q* < 0.05).

The lack of pronounced hematological changes indicates the absence of ongoing systemic inflammation at day 21, whereas the residual changes in the leukocyte differential are consistent with continuing hematopoietic remodeling after injury.

### 2.6. Biodiversity Analysis

**Figure 2:**
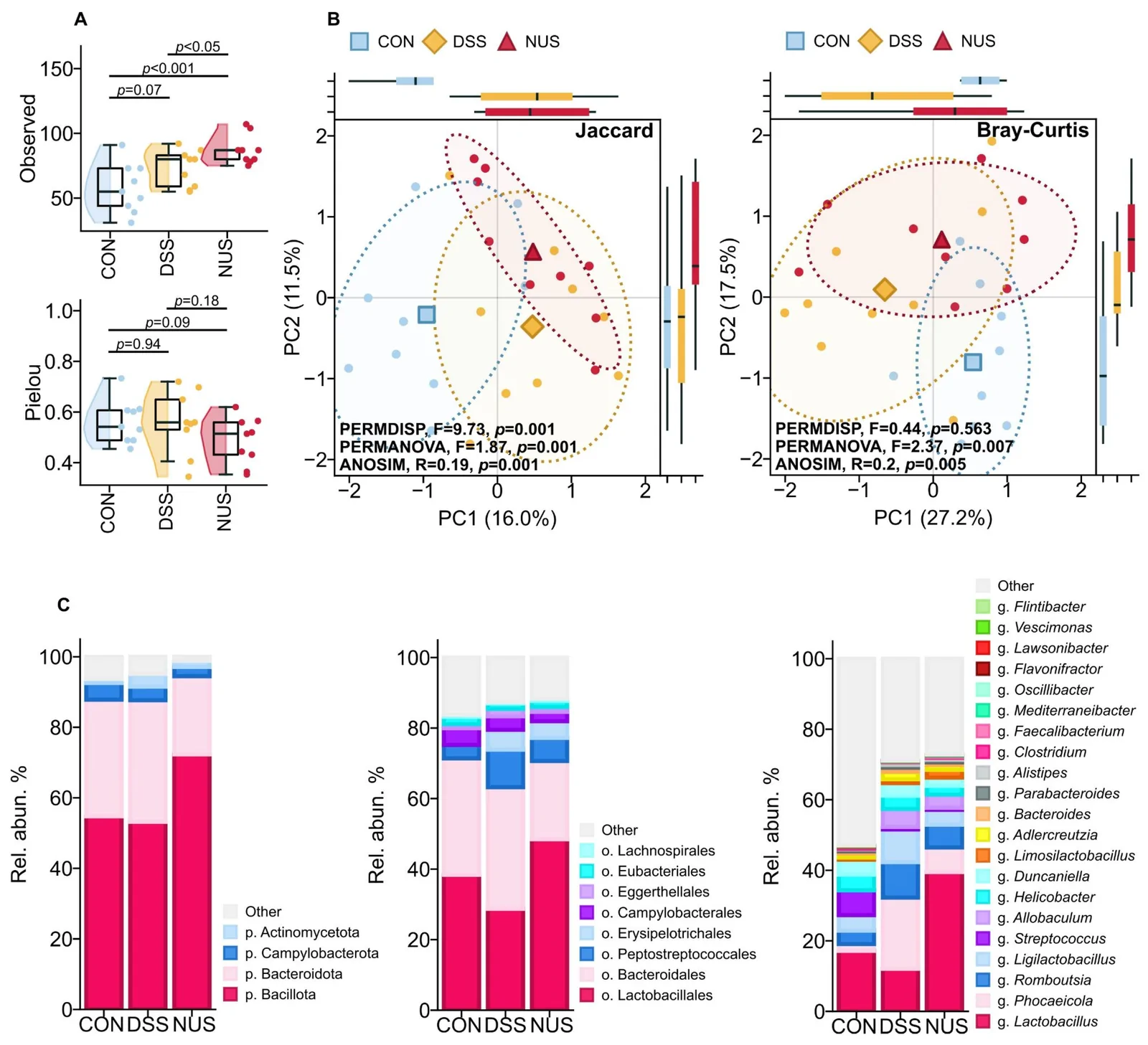
Biodiversity analysis results at the experimental endpoint (day 21) in the unchallenged control group (CON), the DSS-induced colonic injury group receiving standard chow (DSS), and the DSS-induced colonic injury group receiving enteral nutrition (NUS). (**A**) Alpha diversity. Observed species richness (**top**) and Pielou’s evenness index (**bottom**). (**B**) Beta diversity. Principal coordinate ordination (PCoA) based on Jaccard (**left**) and Bray–Curtis (**right**) dissimilarities. Statistical significance of grouping was assessed using PERMANOVA and ANOSIM, each with 999 permutations. (**C**) Relative abundance of the most abundant prevalent phyla, orders, and genera.

Prior to biodiversity analysis, the taxonomic count tables were rarefied to the minimum common depth. Biodiversity analysis revealed significant (*p* < 0.05) differences in within-sample taxonomic richness and community structure between groups (Figure 2A,B).

#### 2.6.1. Alpha Diversity: Species Richness and Evenness

Alpha diversity analysis (Figure 2A) showed an increase in Observed species richness in the DSS group (*p* = 0.07) and especially in the NUS group (*p* < 0.001) compared to the unchallenged control. Median unique species counts were 55.0 (IQR: 44.0; 73.0), 80.0 (IQR: 59.0; 83.0), and 87.0 (IQR: 80.0; 87.0) for CON, DSS, and NUS, respectively. Species evenness (Pielou index) did not differ significantly between groups (*p* > 0.05), though the NUS group showed a trend toward reduced evenness (*p* = 0.09). These results indicate that both injury groups, and particularly the EN group, harbored a greater number of detected species than the unchallenged control, without a corresponding difference in the abundance hierarchy.

#### 2.6.2. Beta Diversity: Structural Dissimilarity of Microbial Communities

Beta diversity was assessed using Jaccard (presence–absence) and Bray–Curtis (abundance-based) dissimilarities (Figure 2B). Both metrics revealed significant separation between groups, as determined by ANOSIM (Jaccard: R = 0.19, *p* = 0.001; Bray-Curtis: R = 0.20, *p* = 0.005). PERMANOVA also detected significant differences among groups (*p* < 0.05). However, heterogeneity of multivariate dispersions was observed for the Jaccard-based analysis (PERMDISP, *p* < 0.05), suggesting that the significant PERMANOVA result may be driven by differences in dispersion rather than by true shifts in community centroids.

Overall, these biodiversity analyses results suggest that the microbial communities of both injury groups differed significantly in composition from that of the unchallenged control at day 21, with the largest difference observed for the NUS group.

#### 2.6.3. Most Abundant Taxonomic Features

The most abundant phyla across all groups were *Bacillota* (formerly *Firmicutes*) and *Bacteroidota* (Figure 2C). *Bacillota* relative abundances were 54.77% (IQR: 45.16; 65.08%), 53.21% (IQR: 39.42; 61.98%), and 72.31% (IQR: 58.94; 77.7%) in CON, DSS, and NUS, respectively; *Bacteroidota* abundances were 33.05% (IQR: 24.44; 40.67%), 34.47% (IQR: 23.25; 45.7%), and 22.18% (IQR: 14.97; 34.8%).

At the order level, the community was dominated by *Lactobacillales*, *Bacteroidales*, *Peptostreptococcales*, *Erysipelotrichales*, and *Campylobacterales*. *Lactobacillales* abundance was 38.36% (IQR: 31.71; 45.99%) in CON, 28.75% (IQR: 20.28; 40.19%) in DSS, and 48.47% (IQR: 34.56; 58.47%) in NUS.

At the genus level, the most abundant taxa included *Lactobacillus*, *Segatella*, *Phocaeicola*, *Ligilactobacillus*, *Romboutsia*, *Duncaniella*, *Streptococcus*, *Allobaculum*, and *Helicobacter*. *Lactobacillus* accounted for 17.16% (IQR: 11.78; 24.22%), 12.08% (IQR: 5.34; 24.55%), and 39.51% (IQR: 24.2; 47.78%) of relative abundance in CON, DSS, and NUS, respectively.

### 2.7. Differential Abundance Analysis

#### 2.7.1. Differential Abundance Analysis of Taxonomic Features

**Figure 3:**
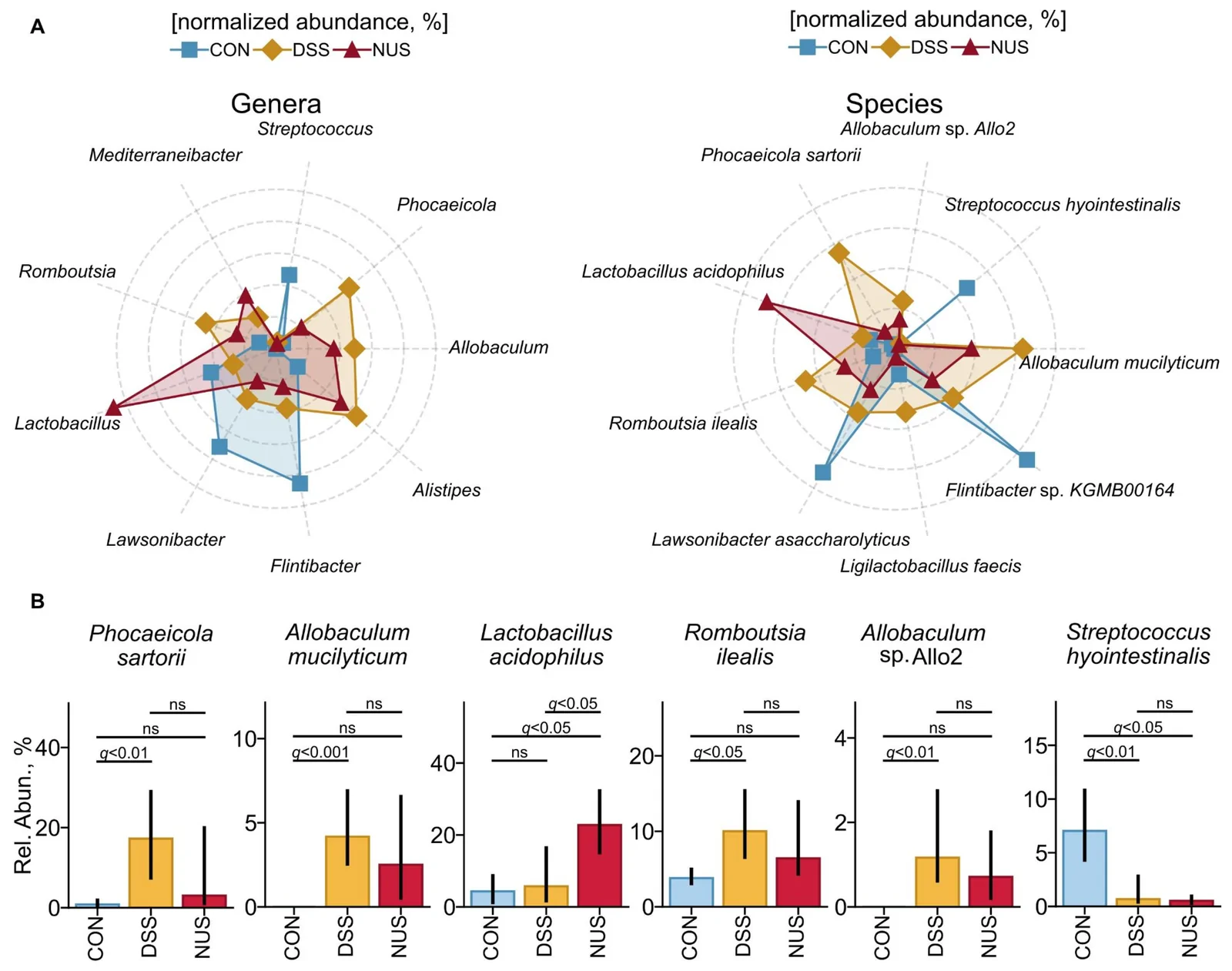
Differentially abundant taxonomic features at the experimental endpoint (day 21) in the unchallenged control group (CON), the DSS-induced colonic injury group receiving standard chow (DSS), and the DSS-induced colonic injury group receiving enteral nutrition (NUS). (**A**) Radar plot of normalized relative abundance of differentially abundant features. (**B**) Relative abundance of differentially abundant features at the species level.

Differential abundance analysis identified two main patterns of taxonomic change (Figure 3A,B). The DSS group showed higher relative abundance of *Bacteroidaceae*, notably *Phocaeicola sartorii*, and of *Erysipelotrichaceae*, notably *Allobaculum mucilyticum*, than the unchallenged control—representing potential dysbiotic elements. The NUS group, in turn, showed higher relative abundance of *Lactobacillus*, with the largest difference observed for *Lactobacillus acidophilus* (Figure 3A).

In particular, the median relative abundance of *Phocaeicola sartorii* reached 17.25% (IQR: 7.45; 28.96%) in the DSS group (*q* < 0.001) and 3.03% (IQR: 1.06; 19.95%) in the NUS group (*q* > 0.05), while it constituted only 0.82% (IQR: 0.11; 1.88%) in the CON group (Figure 3B). Concurrently, *Allobaculum mucilyticum* abundance was 4.17% (IQR: 2.55; 6.90%) in the DSS group (*q* < 0.001) and 2.51% (IQR: 0.53; 6.56%) in the NUS group (*q* > 0.05), whereas it was virtually undetectable in the CON group (0.0% (IQR: 0.0; 0.0%)). The lack of significance for *Phocaeicola* and *Allobaculum* in the NUS group may indicate a weaker difference from the control in this group, although the direction of the difference was the same as in the DSS group. Nevertheless, the observed trend remains consistent with the overall direction of the DSS effect. Finally, *Lactobacillus acidophilus* abundance was 5.72% (IQR: 1.72; 16.39%) in the DSS group, 22.77% (IQR: 15.08; 32.26%) in the NUS group, and 4.33% (IQR: 1.21; 8.66%) in the CON group at the time of measurement, demonstrating a pronounced increase in the NUS group (*q* < 0.05 for CON vs. NUS and DSS vs. NUS).

Higher relative abundance in the DSS group was also observed for *Allobaculum* sp. *Allo2*, *Romboutsia ilealis*, and *Ligilactobacillus faecis*. Notably, *Ligilactobacillus faecis* showed the opposite pattern to *Lactobacillus acidophilus*, with higher abundance in the DSS group (7.85% (IQR: 4.96; 11.69%)) than in both the CON (3.19% (IQR: 2.14; 7.89%), *q* < 0.05) and NUS (1.14% (IQR: 0.53; 3.86%), *q* > 0.05) groups. Furthermore, both DSS-exposed groups showed lower relative abundance of *Streptococcus*, particularly *Streptococcus hyointestinalis* (*q* < 0.05). *Streptococcus hyointestinalis* constituted 7.03% (IQR: 4.31; 10.80%), 0.68% (IQR: 0.42; 2.81%), and 0.52% (IQR: 0.29; 0.95%) of relative abundance in the CON, DSS, and NUS groups, respectively.

#### 2.7.2. Differential Abundance Analysis of Inferred Functional Features

**Figure 4:**
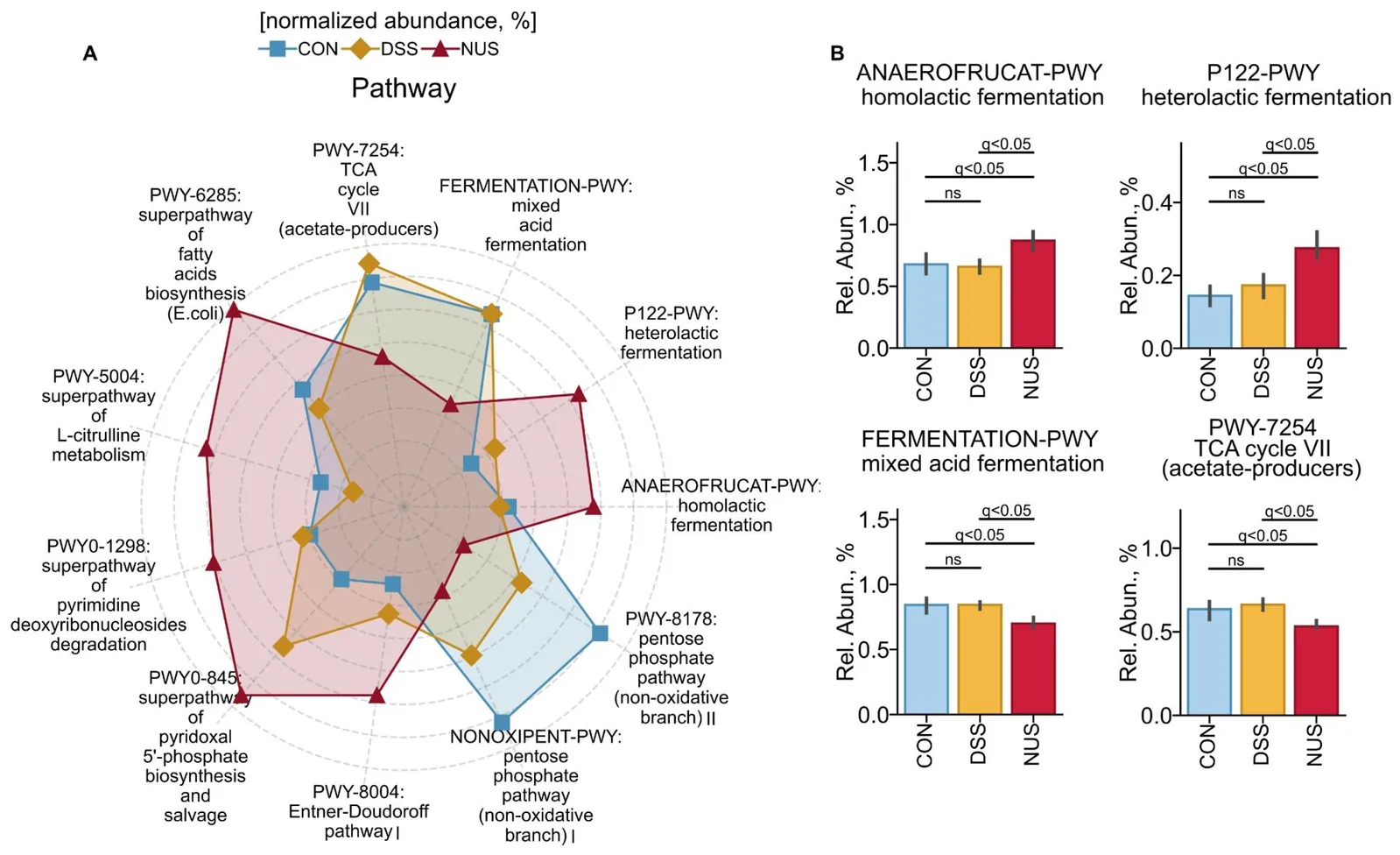
Differentially abundant inferred functional features at the experimental endpoint (day 21) in the unchallenged control group (CON), the DSS-induced colonic injury group receiving standard chow (DSS), and the DSS-induced colonic injury group receiving enteral nutrition (NUS). (**A**) Radar plot of normalized relative abundance of differentially abundant features. (**B**) Relative abundance of differentially abundant features reflecting differences in inferred fermentation capacity between groups.

Pathway analysis revealed differences in inferred fermentation capacity between groups (Figure 4A,B).

In particular, homolactic fermentation (ANAEROFRUCAT-PWY) and heterolactic fermentation (P122-PWY) capacities were higher, whereas mixed-acid fermentation (FERMENTATION-PWY) and the acetate-producing TCA cycle (PWY-7254) were lower in the NUS group than in both CON and DSS (*q* < 0.05, Figure 4B). Collectively, this pattern is consistent with a greater inferred capacity for lactate production relative to acetate production in the NUS group.

Concurrently, the NUS group showed higher inferred potential for several anabolic pathways (Figure 4A), including fatty acid biosynthesis (PWY-6285), L-citrulline metabolism (PWY-5004), pyrimidine deoxyribonucleoside degradation (PWY0-1298), pyridoxal-5′-phosphate biosynthesis and salvage (PWY0-845), and the Entner–Doudoroff pathway (PWY-8004), compared to both CON and DSS (*q* < 0.05). In contrast, the non-oxidative branch of the pentose phosphate pathway capacity appeared to be reduced (NONOXIPENT-PWY), compared to both CON and DSS (*q* < 0.05).

### 2.8. Gene Content of Lactobacillus acidophilus Populations

Given the higher relative abundance of *Lactobacillus acidophilus* in the NUS group, together with the differences in inferred metabolic capacity, we examined whether the *Lactobacillus acidophilus* populations in this group differed in gene content from those in the other groups. To investigate this, we aligned sequencing data to 93 known NCBI strain assemblies and subsequently compared their functional characteristics.

#### 2.8.1. Coverage of *Lactobacillus acidophilus* Reference Genomes

Table 3 shows the relative coverage of reference *Lactobacillus acidophilus* genomes across the experimental groups.

Reference-genome coverage was consistently higher in the NUS group across all reference genomes examined (Table 3). This observation is compatible with either a greater abundance of *Lactobacillus acidophilus* sequence, a broader representation of accessory gene content, or both, and these possibilities cannot be distinguished with the present approach. Across all groups, the microbial community most closely resembled the NCTC13720 strain, with more than 65% genome coverage in all groups. In addition, the NUS group also demonstrated coverage of the NCFM strain, with coverage exceeding 80%. Nonetheless, the current analytical approach does not allow for strain-level resolution, precluding further conclusions.

#### 2.8.2. Differential Abundance Analysis of Functional Clusters in *Lactobacillus acidophilus* Genomes

To characterize gene-content differences within the *Lactobacillus acidophilus* populations between groups, we conducted a differential abundance analysis of functional clusters within the *Lactobacillus acidophilus* pangenome. Homology-based clustering at an 80% identity threshold defined 5850 unique functional clusters, which were then tested for differential representation across experimental groups.

**Figure 5:**
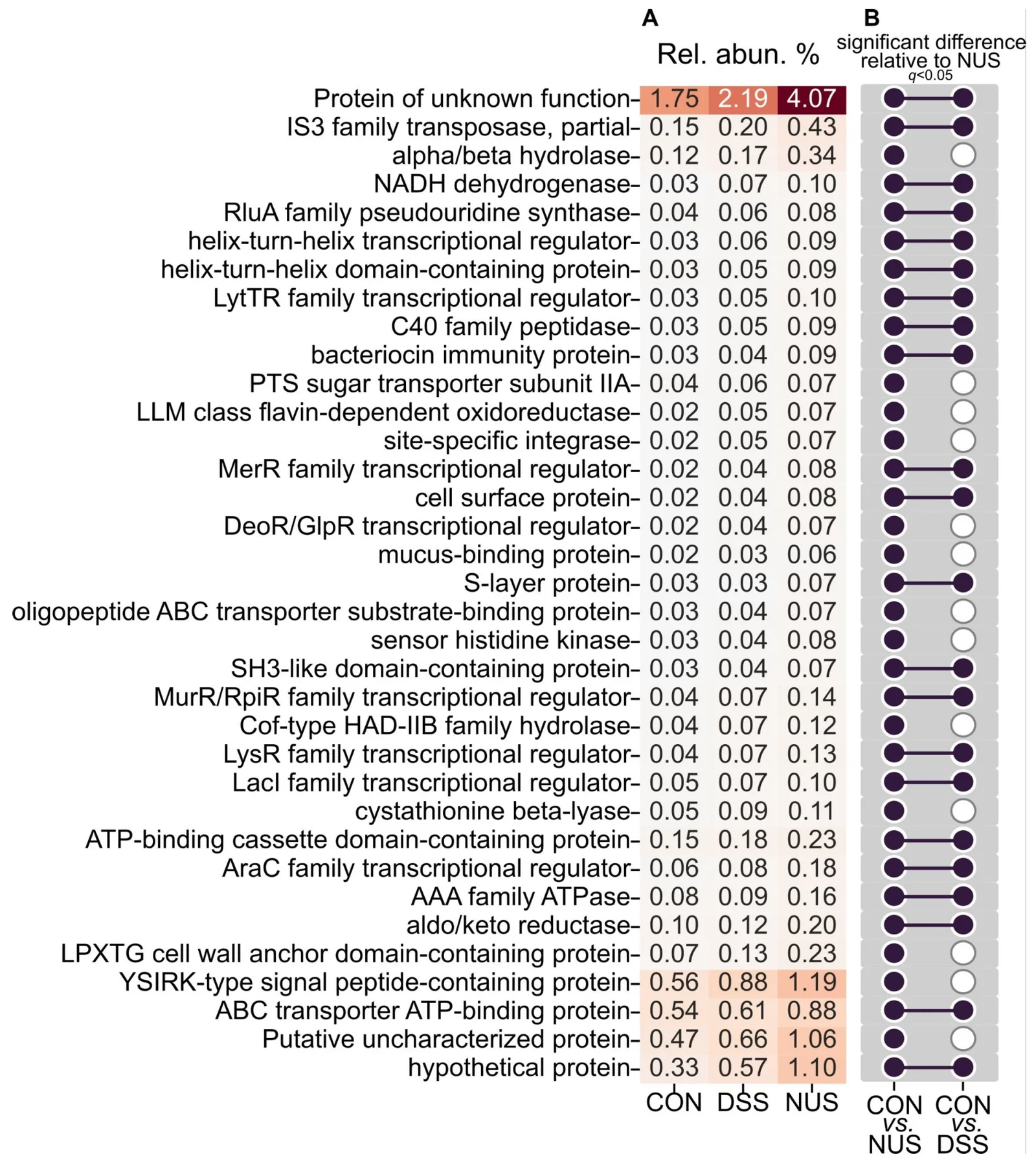
Differentially abundant inferred *Lactobacillus acidophilus* functional clusters at the experimental endpoint (day 21) in the unchallenged control group (CON), the DSS-induced colonic injury group receiving standard chow (DSS), and the DSS-induced colonic injury group receiving enteral nutrition (NUS). (**A**) Heatmap of relative abundance of significantly enriched functional clusters in the NUS samples. Darker colors indicate a higher abundance. (**B**) Set plot depicting the significance of NUS enrichment in pairwise comparisons between CON vs. NUS and DSS vs. CON; filled dark circles denote significant changes (*q* < 0.05).

The relative abundance of 35 functional clusters was higher in the NUS group (Figure 5A,B, *q* < 0.05). Among these, cell surface protein and mucus-binding protein gene copies showed the largest differences (Figure 5A). Cell surface proteins were four-fold more abundant in the NUS genome pool than in CON (*q* < 0.01) and two-fold more abundant than in DSS (*q* < 0.05). Similarly, mucus-binding protein abundance was four-fold higher than in CON (*q* < 0.01) and two-fold higher than in DSS (*q* > 0.05).

Additional enriched clusters included (Figure 5A,B) LPXTG cell wall anchor domain-containing protein, YSIRK-type signal peptide-containing proteins, LacI and AraC family transcriptional regulators, oligopeptide ABC transporter substrate-binding protein, ATP-binding cassette domain-containing protein, S-layer protein, and SH3-like domain-containing protein.

## 3. Discussion

In the present study, we used whole-genome metagenomic sequencing to investigate the effect of enteral nutrition supplementation based on extruded amaranth flour and pea protein on the taxonomic structure of the gut microbiome in a dextran sulfate sodium (DSS) rat model of acute colonic injury. The study utilized three groups, as follows: an unchallenged control group that received standard chow, a colonic injury group that received standard chow, and a colonic injury group that received enteral nutrition.

The present study data suggest that exclusive enteral nutrition administered during the recovery phase following acute colonic epithelial injury promoted accelerated body weight recovery. In particular, acute colonic injury resulted in significant weight loss in experimental animals. However, animals receiving enteral nutrition regained baseline body weight by day 14 post-injury, whereas those in the group on standard chow achieved comparable recovery only by day 21. Nonetheless, daily caloric intake was not controlled, and thus differences in energy intake could not be ruled out as an explanation for the observed weight recovery; further investigation is therefore needed to validate the effect of enteral nutrition on body weight recovery.

Taxonomic biodiversity analysis revealed that the enteral nutrition group showed significantly higher taxonomic richness than the unchallenged control, without a corresponding difference in evenness, indicating greater biodiversity without a marked difference in the existing abundance hierarchy. Furthermore, community composition differed significantly from the unchallenged control in both colonic injury groups, with a larger difference in the group that received enteral nutrition, consistent with two contributing factors, as follows: the injury itself and the dietary intervention.

Further examination revealed that both colonic injury groups showed higher relative abundance of *Bacteroidaceae*, notably *Phocaeicola sartorii*, and *Erysipelotrichaceae*, notably *Allobaculum mucilyticum*, than the unchallenged control—representing potential dysbiotic elements. The enteral nutrition group, in addition, showed higher relative abundance of *Lactobacillus*, with the largest difference observed for *Lactobacillus acidophilus*. Thus, the community differences associated with colonic injury were still present in the enteral nutrition group at day 21, differing from the standard-chow group only in body weight recovery rate and in *Lactobacillales* abundance, but the community composition did not approach that of the unchallenged control.

The higher relative abundance of *Erysipelotrichaceae*, which represented a pronounced taxonomic difference associated with colonic injury in our study, has been previously documented in patients and animal models of inflammatory bowel disease and liver injury [11]. This suggests that the response is reproducible across diverse types of injuries and underscores the need for further investigation into the metabolic capacities of these organisms and the regulatory mechanisms governing their abundance. Furthermore, *Allobaculum mucilyticum* has been specifically implicated in gut barrier dysfunction in mice [12], with pathogenic mechanisms involving disruption of the colonic mucus layer, thereby facilitating intestinal permeability and promoting adhesion of harmful bacteria. In the same study, supplementation with another mucin-degrader, *Akkermansia muciniphila*, was shown to reverse some of these pathogenic changes, raising the question of whether specific enteral nutrition components could be tested to selectively promote beneficial mucin degraders while suppressing pathogenic ones.

Although the potentially dysbiotic mucin degrader remained elevated relative to the unchallenged control in the enteral nutrition group, *Lactobacillus acidophilus* relative abundance in that group was approximately doubled compared to both the unchallenged control and the colonic injury group without enteral nutrition. Evidence from both DSS-induced murine colonic injury models and human IBD patients supports the efficacy of *Lactobacillus* strains in alleviating intestinal damage, reinforcing the intestinal immune barrier, and reducing colitis severity [13,14]. Furthermore, a growing body of evidence indicates that lactate produced by gut bacteria can modulate inflammation via G-protein-coupled receptor 81 (GPR81), and that GPR81 deficiency increases susceptibility to DSS-induced injury [15]. Specifically, lactate has been shown to prevent disruption of tight-junction proteins by inhibiting NF-κB activation through GPR81 in vitro [15]. The higher inferred capacity for lactate-producing fermentation potential and the faster weight recovery observed in our study are consistent with this axis, although establishing such a link would require direct measurement.

In accordance with the taxonomic changes, at the functional level, the group receiving enteral nutrition showed a higher inferred capacity for homolactic and heterolactic fermentation, accompanied by lower mixed-acid fermentation and lower acetate-producing TCA cycle capacity, collectively indicating a greater inferred capacity for lactate production relative to acetate production in this group.

Further analysis of *Lactobacillus acidophilus* gene content across the experimental groups revealed higher recovery of accessory gene clusters in the enteral nutrition group, encoding cell surface proteins and mucin-binding proteins. Such genes underlie adhesive capacity and mucosal association in *Lactobacillus acidophilus* [13,14]. Nevertheless, the current analytical approach precluded more definitive inference.

This study provides novel data on the effects of the enteral nutrition formula based on extruded amaranth flour and pea protein on the gut microbiome after the 21-day period following acute colonic injury. Overall, the observed taxonomic differences align with the composition of the enteral nutrition formula used in this study. Thermomechanical extrusion-cooking gelatinizes the starch matrix and partially converts insoluble dietary fibers into highly soluble fractions. Accordingly, the low caloric density of dietary fiber and the abundance of rapidly fermentable α-glucans are expected to diminish the competitive advantage of fibrolytic *Bacteroidota* on particulate substrates, whereas the vitamin premix and plant-derived peptides are expected to alleviate auxotrophic constraints for saccharolytic lactic acid bacteria requiring B vitamins and specific amino acids. Future investigations should test components individually to broaden our understanding of both individual and synergistic effects.

This study has several limitations. The present study utilized a single-formula enteral nutrition design that does not permit attribution of any observed effects to particular components of the enteral formula (i.e., amaranth, pea protein, or any individual component), limiting understanding of the response to specific enteral components. Dietary fiber content was determined using the AOAC 985.29 method, which accounts only for the high-molecular-weight fraction; therefore, the reported value of 7.7 g/L represents a lower estimate. Energy density was not sufficiently controlled between colonic injury groups due to the multicomponent nature of enteral nutrition, and without accounting for daily caloric intake, the caloric contribution to weight recovery cannot be entirely excluded. The functional profile inferred in this study reflects only genetic potential rather than actual metabolite flux, as follows: concentrations of short-chain fatty acids and lactate were not measured. Furthermore, neither GPR81 expression nor epithelial repair markers were measured. Moreover, functional inference was performed on classified reads only, so it describes the classifiable fraction of the community. This fraction did not differ between groups, so the between-group comparisons are not expected to be differentially biased, but the absolute capacities should be interpreted with corresponding caution. Additionally, histological assessment was performed only at day 3 to verify the injury model; no endpoint histology or markers of epithelial repair were assessed, so no direct conclusion regarding mucosal healing can be drawn. Finally, the analysis of *Lactobacillus acidophilus* gene content was based on read recruitment to reference genomes and pangenome clusters; thus, this approach characterizes recovered gene content but does not resolve strains.

## 4. Materials and Methods

### 4.1. Animals and Study Design

This study was performed on 30 outbred Wistar rats of both sexes (15 females, 15 males), aged 3 months at the start of the experiment, obtained from the vivarium of Astana Medical University (Astana, Kazakhstan). Median baseline body weight was 280.4 (IQR: 234.0; 335.25) g and did not differ between groups (CON 263.0 (IQR: 219.0; 324.0) g; DSS 334.0 (IQR: 234.5; 336.5) g; NUS 280.4 (IQR: 234.5; 302.0) g; *p* = 0.85, Kruskal–Wallis test). Baseline weight differed by sex (females 234.5 (IQR: 216.8; 256.5) g; males 335.25 (IQR: 307.5; 377.1) g); sex was balanced across groups (CON: 5F/4M; DSS: 4F/5M; NUS: 4F/5M). Twenty-seven animals (13 females, 14 males) were allocated to three groups of nine by simple randomization after stratification by baseline body weight and sex, using a random number sequence generated in Microsoft Excel: CON (unchallenged control, *n* = 9)—received drinking water for 3 days, followed by standard chow for 21 days; DSS (injury without intervention, *n* = 9)—received 5% DSS for 3 days, followed by standard chow for 21 days; NUS (injury and enteral feeding intervention, *n* = 9)—received 5% DSS for 3 days, followed by the enteral nutrition formula as the sole caloric source for 21 days. Three additional animals (2 females, 1 male) received 5% DSS in parallel and were euthanized on day 3 for histological verification of the model (Section 4.2); these animals were not part of the CON/DSS/NUS cohorts and were excluded from all endpoint analyses. The experimental unit was the individual animal. No animals died or were withdrawn during the experiment. Investigators were not blinded to group allocation. The full animal flow, including group allocation and the sample size used for each analysis, is shown in Figure 1.

The unchallenged control was not subjected to injury and represents the normal status. The appropriate comparison for evaluating the efficacy of the enteral feeding intervention is between the DSS and NUS groups. Body weight was recorded on days 1, 3, 8, 14, and 21. At the end of the experiment, animals were euthanized by carbon dioxide overdose [16]. The study was approved by the Local Bioethics Committee at the National Laboratory Astana, Nazarbayev University (Protocol Number 04-2023, date 8 November 2023, Astana, Kazakhstan) and was conducted in accordance with Directive 2010/63/EU on the protection of animals used for scientific purposes, the applicable national legislation of the Republic of Kazakhstan on the humane treatment of laboratory animals, and the institutional guidelines for animal care and use of Nazarbayev University. Reporting follows the ARRIVE 2.0 guidelines. The full animal flow, including group allocation and the sample size used for each analysis, is shown in Figure 1.

### 4.2. Histological Examination

To verify the DSS acute colonic injury model, three additional DSS-challenged animals were euthanized on day 3 of the experiment. From each animal, six intestinal tissue fragments measuring 0.5–1.0 cm in length were collected at equidistant intervals, with the anatomical section (proximal, middle, distal) recorded. Samples were fixed in 10% formalin at 4 °C for at least 24 h, washed with water, dehydrated through a graded series of increasing ethanol concentrations (70%, 90%, 95%, 100%), cleared in xylene, and embedded in paraffin. Sections of 5 µm thickness were prepared using a Leica SM 2000R sliding microtome (Leica Biosystems Nussloch, Nußloch, Germany), deparaffinized in xylene, and rehydrated through a graded series of decreasing ethanol concentrations (100%, 96%, 70%).

Histopathological and morphometric analyses were performed on both transverse and longitudinal sections; morphometric measurements were conducted in 10 fields of view. The severity of injury was assessed using the Mouse Colitis Histology Index (MCHI) [10], which is calculated as the sum of scores across four criteria, as follows: crypt hyperplasia, crypt density, inflammatory infiltration, and number of crypt abscesses and ulcerations. Microscopy was performed using the CELENA^®^ X High Content Imaging System (Logos Biosystems, Anyang-si, Republic of Korea); three sections per animal were analyzed, each in triplicate.

### 4.3. Preparation of the Enteral Nutrition Mixture

A 500 mL batch of the enteral mixture contained the following components: extruded fine amaranth flour—200.0 g; pea protein isolate—21.5 g; safflower oil—30.0 g; maltodextrin—10.0 g; 0.05% aqueous solution of sunflower lecithin—25.0 mL; carrageenan—0.30 g; sodium ascorbate—0.25 g; vitamin premix—0.19 g; mineral premix—1.65 g; deionized water—q.s. to 500 mL. The energy density was 2.23 kcal/mL.

The dry phase (flour, pea protein, maltodextrin, carrageenan, sodium ascorbate, and premixes) was homogenized in a laboratory mixer. The lipid phase was prepared by dissolving lecithin in deionized water, adding safflower oil, and processing with a T 25 digital ULTRA-TURRAX^®^ (IKA-Werke, Staufen, Germany) at 9500 rpm for 4 min. The dry mixture was introduced into the emulsion at 500 rpm, the volume was brought to 500 mL, and the mixture was subjected to final colloidal homogenization.

### 4.4. Determination of Enteral Nutrition Dietary Fiber Content

The dietary fiber content in the final formulation was determined using the enzymatic-gravimetric method AOAC 985.29 (AACC 32-05.01). Samples were sequentially digested in phosphate buffer (pH 6.0) with thermostable α-amylase, protease, and amyloglucosidase. The residue was precipitated with 95% ethanol, washed with ethanol and acetone, filtered, dried, and weighed; the mass was corrected for ash (AOAC 942.05) and protein content. Total dietary fiber content was 7.7 g/L (0.34 g per 100 kcal). This method quantifies the high-molecular-weight fraction; low-molecular-weight soluble oligosaccharides are not included in the analysis.

### 4.5. Hematological Analysis

Blood for hematological analysis was collected at the experimental endpoint (day 21) terminally by cardiac puncture under anesthesia immediately prior to euthanasia. Samples were collected into single-use polypropylene tubes containing EDTA-K2 anticoagulant (BD Vacutainer^®^ K2EDTA Blood Collection Tubes; Becton, Dickinson and Company (BD), Franklin Lakes, NJ, USA) and stored at room temperature for no more than 4 h prior to analysis, with thorough mixing with the anticoagulant ensured by gentle inversion.

Complete blood count was performed using an automatic veterinary hematology analyzer (Rayto RT-7600 Veterinary Auto Hematology Analyzer; Rayto Life and Analytical Sciences Co., Shenzhen, China) according to the manufacturer’s instructions, with instrument calibration performed using control materials prior to each measurement series. The following 20 hematological parameters were determined: white blood cell count (WBC, ×10^9^/L), lymphocyte count (LYM#, ×10^9^/L), monocyte/middle cell count (MID#, ×10^9^/L), granulocyte count (GRA#, ×10^9^/L), lymphocyte percentage (LYM%, %), monocyte/middle cell percentage (MID%, %), granulocyte percentage (GRA%, %), red blood cell count (RBC, ×10^12^/L), hemoglobin concentration (HGB, g/L), hematocrit (HCT, %), mean corpuscular volume (MCV, fL), mean corpuscular hemoglobin (MCH, pg), mean corpuscular hemoglobin concentration (MCHC, g/L), standard deviation of red cell distribution width (RDW-SD, fL), coefficient of variation in red cell distribution width (RDW-CV, %), platelet count (PLT, ×10^9^/L), mean platelet volume (MPV, fL), platelet distribution width (PDW, %), platelet–large cell ratio (P-LCR, %), and platelet crit (PCT, %). Each sample was analyzed in triplicate; the mean value of the replicates was used for subsequent analysis.

### 4.6. DNA Extraction and Metagenomic Sequencing

Genomic DNA was isolated using the ZymoBIOMICS DNA Miniprep Kit (Cat# D4300, Zymo Research, Irvine, CA, USA) in strict accordance with the manufacturer’s instructions. The quality, purity, and structural integrity of the extracted DNA were comprehensively evaluated using complementary analytical platforms, as follows: spectrophotometric ratios (A_260_/A_280_ and A_260_/A_230_) were determined via a NanoDrop 2000 spectrophotometer (Thermo Fisher Scientific, Waltham, MA, USA), total DNA concentration was measured using a Qubit 3.0 fluorometer (Thermo Fisher Scientific, Waltham, MA, USA), and molecular weight integrity was verified by agarose gel electrophoresis. To monitor and control for potential reagent-derived contamination, extraction-negative controls were processed in parallel with each batch and subjected to downstream sequencing.

Library preparation and high-throughput shotgun metagenomic sequencing were executed by BGI Tech Solutions Co., Ltd. (Hong Kong, China) on the DNBSEQ platform utilizing a paired-end configuration (2 × 150 bp). To ensure robust coverage and taxonomic resolution of low-abundance microbial taxa, a minimum sequencing depth of 6 Gb per sample was enforced.

All of the 27 samples were successfully sequenced. Each sample generated a median of 48.36 (IQR: 48.21; 48.41; range: 48.2–48.5), 48.29 (IQR: 48.27; 48.38; range: 48.2–48.5), and 48.29 (IQR: 48.16; 48.35; range: 34.9–48.5) million reads for the CON, DSS, and NUS groups, respectively (*p* = 0.64, Kruskal–Wallis test).

### 4.7. Taxonomic Profiling, Functional Inference, and Strain-Level Alignments

#### 4.7.1. Taxonomic Profiling

Read quality was assessed using Fastp v0.23.4 [17], and adapter sequences and low-quality bases were trimmed. Bases were required to have a minimum quality score of Q30, with a tolerance of 40%. Additionally, reads with trimmed lengths shorter than 35 bp were filtered out. Fastp analysis indicated that 95.19% (IQR: 94.78; 95.54), 94.58% (IQR: 94.58; 94.88), and 94.83% (IQR: 94.48; 94.99) of reads were retained for the CON, DSS, and NUS groups, respectively (*p* = 0.12, Kruskal–Wallis test).

Subsequently, potential contamination was removed using Hostile v2.0.2 [18] in paired-end/bowtie2 mode against the T2T-CHM13v2.0 (*Homo sapiens*) and GRCr8 (*Rattus norvegicus*) reference genomes. This removed a median of 0.04% (IQR: 0.02; 0.12), 0.06% (IQR: 0.03; 0.10), and 0.04% (IQR: 0.03; 0.06) of reads aligning to the human genome (*p* = 0.87, Kruskal–Wallis test), and 6.76% (IQR: 3.19; 17.97), 9.23% (IQR: 4.90; 15.68), and 5.96% (IQR: 4.34; 9.41) of reads aligning to the rat genome (*p* = 0.84, Kruskal–Wallis test). Finally, a median of 42.95 (IQR: 37.87; 44.65), 42.02 (IQR: 38.94; 43.48), and 42.09 (IQR: 34.76; 43.81) million reads were retained for downstream analysis (*p* = 0.73, Kruskal–Wallis test).

After initial filtration, Kraken2 v2.17.1 [19] was used to annotate reads against the precompiled Standard-16 database index (dated 26 June 2026). Taxonomic classification was performed with a confidence threshold of 0.05 and a minimum group hits threshold of 3 in paired-end read mode. Additionally, the -report-minimizer-data flag was used to report the number of total and unique k-mers mapped per reference genome, and the -classified-out flag was used to retain only classified reads for downstream analyses. In total, the proportion of successfully classified reads was 10.1% (IQR: 5.63; 16.49%), 16.59% (IQR: 6.03; 21.06%), and 12.87% (IQR: 7.50; 13.80%) for the CON, DSS, and NUS groups, respectively (*p* = 0.61, Kruskal–Wallis test).

The ETE3 v3.1.3 utility [20] was used for taxonomic nomenclature resolution based on NCBI taxonomy IDs.

Kraken2 metagenomic assignments operate at the k-mer level—specifically, the tool splits reads into k-mers and maps them against k-mers in the reference genomes database. To quantify taxonomic abundance, we first estimated reference genome coverage. Specifically, reference coverage (C) was calculated as the ratio of unique k-mers mapped to a reference genome to the total number of unique k-mers in that reference genome. We then assessed the quality of taxonomic assignments using an E-value filter approach, as described in [21]. The E-value was calculated as (K/R) × C, where K is the number of unique k-mers assigned to a taxon, R is the number of reads assigned to that taxon, and C is the k-mer coverage. This composite metric penalizes assignments where many reads map to only a few unique k-mers (lower K/R) or where the covered k-mers are not well distributed across the genome (lower C). Only assignments with an E-value score of >0.15 were retained for downstream analysis. Finally, to account for differences in reference genome sizes, the total number of assigned reads was divided by the number of unique k-mers in each reference genome, and relative abundance was calculated using TSS (total sum scaling).

#### 4.7.2. Functional Inference

Following taxonomic classification, the HUMAnN [22] v4.0.0.alpha.2 pipeline was used to estimate the functional capacity of classified reads against the EC-filtered UniRef90 database (https://github.com/biobakery/humann, accessed on 8 July 2026). Since taxonomic classification had already been performed with Kraken2, the HUMAnN taxonomic profiling step was omitted, and the pipeline proceeded directly to the BLASTX alignment step using the following parameters: -bypass-nucleotide-index, -bypass-nucleotide-search, and -bypass-prescreen. Translated alignment was performed with a minimum identity threshold of 30% and a minimum query coverage threshold of 80%. Functional annotations were quantified against the MetaCyc pathway database. The resulting annotations were quantified in counts per million (CPM) units, aggregated to retain only classified functions, and normalized using TSS.

#### 4.7.3. Strain-Level Alignment

Finally, the BV-BRC database [23] was used to compile a list of all *Lactobacillus acidophilus* (BV-BRC taxon ID 1579) NCBI assemblies. Subsequently, the NCBI dataset [24] utility v18.33.1 was used to download all available *Lactobacillus acidophilus* genomes and protein annotations that passed CheckM-reported completeness of >90% and exhibited contamination of <5%. In total, 93 unique *Lactobacillus acidophilus* strain assemblies, comprising 175,836 unique proteins, were downloaded.

Next, MMseqs2 [25] v18.8cc5c was used to cluster *Lactobacillus acidophilus* proteins into functional clusters for downstream analysis. MMseqs2 was run in “easy-cluster” mode with a minimum query and subject identity threshold of 80%. MMseqs2 analysis yielded 5850 unique 80% identity clusters, of which 1229 clusters contained at least five homologous sequences. For cluster annotation, the most common canonized nomenclature reported by the NCBI dataset was used.

DIAMOND [26] aligner v2.1.9 was then used to create a BLASTX database for these genomes, and preprocessed reads were annotated against this database with thresholds of 80% identity, a bit score of 50, and an e-value of 10^−5^. Only the best match per read was retained. Genome coverages were then estimated as the ratio of recovered proteins to the total number of unique proteins in each genome. Finally, to estimate the relative abundance of each *Lactobacillus acidophilus* functional cluster across samples, total read counts were calculated for each cluster per sample, normalized by subject length, and further normalized using TSS.

All steps of the preprocessing pipeline 4.7.1.–4.7.3. can be found at GitHub: https://github.com/VeaLi/microbiome-analysis-scripts-enteral-nutrition-colonic-injury-jul-2026 (accessed on 31 July 2026).

### 4.8. Statistical Analysis

Statistical analyses were performed in Python v3.12.2 using NumPy v1.26.4 [27], SciPy v1.11.4 [28], statsmodels v0.14.6 [29], and pandas v3.0.1 [30]. Biodiversity analyses were conducted with scikit-bio v0.6.3 [31], and visualizations were prepared using Matplotlib v3.10.8 [32] and seaborn v0.13.2 [33].

Weight data were compared between groups and between time points. *Omnibus* testing was performed using either the Kruskal–Wallis H-test or the Friedman test. *Post hoc* comparisons were conducted using either the Mann–Whitney U test or the Wilcoxon test, as appropriate. Test results were adjusted using the Benjamini-Hochberg (BH) FDR procedure. Differences were considered significant at *q* < 0.05.

Hematological data were compared across three hypothesis families, as follows: leukocytes, erythrocytes, and thrombocytes. *Omnibus* testing was performed using either one-way ANOVA or the Kruskal–Wallis H-test, depending on data distribution. *Post hoc* comparisons were conducted using either the *t*-test or the Mann–Whitney U test, as appropriate. Normality of the data was assessed using the Shapiro–Wilk test, and equality of variances was evaluated using Levene’s test. *Omnibus* and *post hoc* test results were adjusted using the Benjamini-Hochberg (BH) FDR procedure. Differences were considered significant at *q* < 0.05.

For biodiversity analyses, the data were rarefied to the minimum common depth. Beta diversity was compared using Jaccard and Bray–Curtis distances. The significance of group separation was evaluated using ANOSIM and PERMANOVA, both with 999 permutations. Prior to PERMANOVA, homogeneity of multivariate dispersions was assessed using PERMDISP. Alpha diversity was assessed using the Observed richness index and Pielou’s evenness index. Pairwise comparisons were performed using the independent *t*-test or the Mann–Whitney U test, depending on data distribution.

Differential analysis was performed using relative abundance data as follows: first, comparisons were pre-filtered using distribution-free two-sample Hodges–Lehmann estimator confidence intervals as described in [34], which retains only differences with visually separable effects. Next, pre-filtered hypotheses were adjusted using the FDR–BH procedure. Differences were considered significant if they had non-overlapping confidence intervals for the difference between medians and an FDR–BH-adjusted *q*-value < 0.05.

## 5. Conclusions

In a rat model of acute colonic injury, exclusive enteral nutrition during recovery was associated with faster body weight regain than standard chow. However, at day 21, the gut microbial community of both colonic injury groups was distinct from that of the unchallenged control. In particular, both injury groups showed higher relative abundances of *Bacteroidaceae* and *Erysipelotrichaceae*, notably the mucin-degrader *Allobaculum mucilyticum*. Nonetheless, the enteral nutrition group showed higher relative abundance of *Lactobacillus* compared to both the unchallenged control and the injury group on standard chow, with the largest difference observed for *Lactobacillus acidophilus*, as well as a greater inferred capacity for lactate-producing fermentation.

Collectively, these physiological, taxonomic, and functional data are consistent with a role for community metabolic output in the effects of enteral nutrition, with lactobacilli as one of the potential mediators. Future studies should include direct measurements of short-chain fatty acids and lactate, evaluate the individual and synergistic effects of formula components (e.g., via an isocaloric amaranth-free control), and explore novel components aimed at selectively promoting beneficial mucin degraders and lactate-producing bacteria.

## Figures and Tables

**Table 1:** Body weight dynamics (% of baseline) in the unchallenged control group (CON), the DSS-induced colonic injury group receiving standard chow (DSS), and the DSS-induced colonic injury group receiving enteral nutrition (NUS). Data are presented as median and interquartile range (IQR).

Parameter	CON, *n* = 9	DSS, *n* = 9	NUS, *n* = 9	Q-Value (Between Groups)
Day 3	101.69 (101.2; 102.28)	95.17 (94.76; 95.51)	97.51 (97.22; 97.72)	*q* < 0.0001 ^a^
Day 8	103.88 (103.7; 105.59)	94.29 (93.41; 95.13)	93.19 (92.8; 93.61)	*q* < 0.0001 ^a^
Day 14	112.55 (108.82; 116.73)	94.91 (92.99; 97.47)	101.78 (101.58; 101.93)	*q* < 0.0001 ^a^
Day 21	127.73 (118.83; 143.2)	100.51 (97.1; 102.53)	110.1 (109.91; 110.91)	*q* < 0.0001 ^a^
**Q-value (within groups)**	*q* < 0.0001 ^b^	*q* < 0.05 ^b^	*q* < 0.0001 ^b^	**-**

^a^ Kruskal–Wallis H-test; ^b^ Friedman test.

**Table 2:** Hematological parameters at the experimental endpoint (day 21) in the unchallenged control group (CON), the DSS-induced colonic injury group receiving standard chow (DSS), and the DSS-induced colonic injury group receiving enteral nutrition (NUS). Data are presented as median and interquartile range (IQR).

Parameter	CON, *n* = 9	DSS, *n* = 9	NUS, *n* = 9	Q-Value
**While Blood Cell count**				
WBC (×10^9^/L)	5.85 (4.86; 6.68)	5.62 (3.96; 6.3)	7.39 (4.46; 9.23)	*q* = 0.39 ^a^
LYM# (×10^9^/L)	3.08 (2.9; 3.26)	1.53 (1.31; 2.45)	2.79 (1.88; 2.82)	*q* < 0.05 ^a^
LYM% (%)	46.8 (39.1; 55.7)	35.7 (28.6; 41.6)	35.6 (31.6; 38.2)	*q* < 0.05 ^b^
GRA# (×10^9^/L)	2.67 (2.36; 3.17)	2.89 (2.61; 3.5)	4.08 (3.83; 5.73)	*q* = 0.13 ^a^
GRA% (%)	48.2 (40.3; 55.6)	59.7 (53.2; 66.0)	60.2 (55.2; 63.3)	*q* < 0.05 ^b^
MID# (×1010^9^/L)	0.26 (0.23; 0.35)	0.27 (0.21; 0.32)	0.49 (0.35; 0.51)	*q* = 0.18 ^a^
MID% (%)	5.0 (4.0; 5.3)	5.2 (5.0; 5.7)	5.7 (4.9; 6.6)	*q* = 0.35 ^b^
**Red Blood Cell count**				
RBC (×10^12^/L)	5.06 (4.71; 5.36)	5.01 (4.55; 5.61)	4.67 (4.42; 4.9)	*q* = 0.84 ^a^
HGB (g/L)	117.0 (106.0; 119.0)	114.0 (110.0; 116.0)	107.0 (103.0; 109.0)	*q* = 0.65 ^a^
HCT (%)	28.5 (26.9; 29.6)	28.4 (27.3; 31.0)	26.8 (26.1; 27.3)	*q* = 0.65 ^a^
MCV (fL)	56.3 (54.3; 57.2)	56.7 (55.3; 57.6)	57.0 (54.8; 58.2)	*q* = 0.92 ^b^
MCHC (g/L)	409.0 (376.0; 418.0)	393.0 (387.0; 420.0)	395.0 (392.0; 403.0)	*q* = 0.92 ^b^
MCH (pg)	22.8 (21.0; 24.2)	22.0 (21.4; 24.8)	22.2 (22.0; 23.0)	*q* = 0.92 ^b^
RDW-SD (fL)	36.9 (36.0; 37.3)	36.3 (36.0; 38.9)	38.0 (35.4; 39.0)	*q* = 0.92 ^b^
RDW-CV (%)	14.3 (14.2; 15.1)	14.5 (14.4; 14.7)	15.0 (14.1; 15.2)	*q* = 0.92 ^b^
**Platelet count**				
PLT (×10^9^/L)	539.0 (361.0; 562.0)	535.0 (510.0; 582.0)	616.0 (578.0; 672.0)	*q* = 0.09 ^a^
MPV (fL)	6.4 (6.3; 6.5)	6.4 (6.3; 6.6)	6.2 (6.0; 6.3)	*q* = 0.13 ^b^
PDW (%)	12.3 (11.3; 13.0)	11.2 (10.0; 11.7)	10.5 (10.0; 11.0)	*q* = 0.13 ^a^
P-LCR (%)	3.4 (3.0; 5.9)	6.4 (2.7; 8.4)	2.1 (1.8; 2.6)	*q* < 0.05 ^a^
PCT (%)	0.34 (0.24; 0.36)	0.34 (0.31; 0.36)	0.39 (0.36; 0.41)	*q* = 0.13 ^a^

^a^ Kruskal–Wallis H-test; ^b^ one-way ANOVA.

**Table 3:** Genome coverage (%) of *Lactobacillus acidophilus* strains at the experimental endpoint (day 21) in the unchallenged control group (CON), the DSS-induced colonic injury group receiving standard chow (DSS), and the DSS-induced colonic injury group receiving enteral nutrition (NUS). Data are presented as median and interquartile range (IQR).

*Lactobacillus acidophilus* Strain	CON, *n* = 9	DSS, *n* = 9	NUS, *n* = 9
NCTC13720	73.57 (58.4; 77.02)	67.98 (62.22; 74.57)	78.31 (76.67; 79.57)
NCFM	49.38 (40.49; 62.97)	65.36 (56.31; 72.69)	83.89 (80.64; 84.99)
30SC	34.82 (27.46; 41.14)	36.28 (29.43; 42.28)	48.76 (45.9; 52.11)
NCTC13721	37.2 (29.3; 45.1)	30.9 (23.68; 39.31)	48.85 (41.96; 55.56)
CIRM-BIA 444	16.9 (9.64; 26.8)	29.4 (20.03; 34.82)	44.61 (42.45; 46.42)
YT1	11.27 (6.95; 17.98)	19.0 (12.99; 22.32)	30.16 (28.12; 31.47)
ATCC 4796	10.69 (7.18; 15.94)	17.13 (12.4; 19.95)	25.79 (24.36; 26.83)
NCTC1407	13.58 (11.85; 14.71)	15.57 (13.33; 16.28)	16.36 (15.44; 18.32)

## Data Availability

Sequence data of the current study have been deposited in the NCBI BioProject; the primary accession number is PRJNA1499926. All steps of the taxonomic profiling and functional inference can be found at GitHub: https://github.com/VeaLi/microbiome-analysis-scripts-enteral-nutrition-colonic-injury-jul-2026 (accessed on 31 July 2026).

## Abbreviations

The following abbreviations are used in this manuscript:

DSS	Dextran sulfate sodium
EN	Enteral nutrition
IBD	Inflammatory bowel disease
TSS	Total sum scaling
